# Diabetes MILES – The Netherlands: rationale, design and sample characteristics of a national survey examining the psychosocial aspects of living with diabetes in Dutch adults

**DOI:** 10.1186/1471-2458-12-925

**Published:** 2012-10-30

**Authors:** Giesje Nefs, Mariska Bot, Jessica L Browne, Jane Speight, François Pouwer

**Affiliations:** 1 Department of Medical and Clinical Psychology, Center of Research on Psychology in Somatic diseases (CoRPS), Tilburg University, PO BOX 90153 5000, LE, Tilburg, The Netherlands; 2 Department of Psychiatry, VU University Medical Center and GGZ in Geest, Amsterdam, The Netherlands; 3 The Australian Centre for Behavioural Research in Diabetes, Diabetes Australia – Vic, Melbourne, VIC, Australia; 4 Centre for Mental Health and Wellbeing Research, School of Psychology, Deakin University, Burwood, VIC, Australia; 5 AHP Research, Hornchurch, Essex, UK

**Keywords:** Diabetes, Psychology, Self-care, Well-being, National survey

## Abstract

**Background:**

As the number of people with diabetes is increasing rapidly worldwide, a more thorough understanding of the psychosocial aspects of living with this condition has become an important health care priority. While our knowledge has grown substantially over the past two decades with respect to the physical, emotional and social difficulties that people with diabetes may encounter, many important issues remain to be elucidated. Under the umbrella of the Diabetes MILES (Management and Impact for Long-term Empowerment and Success) Study International Collaborative, Diabetes MILES – The Netherlands aims to examine how Dutch adults with diabetes manage their condition and how it affects their lives. Topics of special interest in Diabetes MILES - The Netherlands include subtypes of depression, Type D personality, mindfulness, sleep and sexual functioning.

**Methods/design:**

Diabetes MILES – The Netherlands was designed as a national online observational study among adults with diabetes. In addition to a main set of self-report measures, the survey consisted of five complementary modules to which participants were allocated randomly. From September to October 2011, a total of 3,960 individuals with diabetes (40% type 1, 53% type 2) completed the battery of questionnaires covering a broad range of topics, including general health, self-management, emotional well-being and contact with health care providers. People with self-reported type 1 diabetes (specifically those on insulin pump therapy) were over-represented, as were those using insulin among respondents with self-reported type 2 diabetes. People from ethnic minorities were under-represented. The sex distribution was fairly equal in the total sample, participants spanned a broad age range (19–90 years), and diabetes duration ranged from recent diagnosis to living with the condition for over fifty years.

**Discussion:**

The Diabetes MILES Study enables detailed investigation of the psychosocial aspects of living with diabetes and an opportunity to put these findings in an international context. With several papers planned resulting from a pooled Australian-Dutch dataset and data collections planned in other countries, the Diabetes MILES Study International Collaborative will contribute substantially to identifying potentially unmet needs of those living with diabetes and to inform clinical research and care across the globe.

## Background

Diabetes mellitus is affecting a growing number of people worldwide. Global prevalence estimates of this chronic metabolic condition are projected to rise from 171 million in 2000 to 366 million in 2030 [1]. Given the associated increased risk of disability [2,3] and mortality [4], diabetes is considered one of the main threats to human health of the 21^st^ century [5]. In The Netherlands, the scope of the problem is similar to these global trends, with approximately 750,000 people having a diagnosis of diabetes [6]. In high-income countries, type 2 diabetes accounts for about 85 to 95% of all diabetes cases [7]. Although less prevalent, type 1 diabetes represents a high burden of co-morbidities and costs, as people with type 1 diabetes generally live with their condition for a longer period of time, and may develop complications at an earlier stage of life [8,9].

### Living with diabetes

Leading a fulfilling and enjoyable life while having diabetes is certainly possible for many people but, for others, coping with the condition, its management and its complications can be both demanding and challenging. Diabetes can be accompanied by distressing acute short-term complications, such as hypoglycaemia and ketoacidosis, and by long-term micro-vascular complications (e.g. retinopathy, neuropathy and nephropathy) and macro-vascular disease (e.g. stroke, heart disease) [10]. From previous ground-breaking studies, we know that these vascular conditions can be prevented or delayed through optimal management of blood glucose levels and other cardiovascular risk factors [11-13]. The importance of daily self-management is therefore unquestionable but can place a heavy burden on individuals. Self-management encompasses a wide range of activities, including daily oral medication and/or insulin use, blood glucose monitoring, foot care, healthy eating (and, for some, carbohydrate counting), and engaging in regular physical activity. Unsurprisingly, diabetes can have a serious impact on the emotional well-being and quality of life of people living with the condition [14-17]. In turn, emotional distress may hamper self-care behaviours and increase the risk of adverse diabetes outcomes [18,19]. While our understanding of the psychosocial aspects of diabetes has increased enormously in the past twenty years [20-24], many important issues remain to be elucidated.

### The Diabetes MILES Study International Collaborative

The Diabetes MILES (Management and Impact for Long-term Empowerment and Success) Study has been established as an international collaborative, involving a series of national surveys and cohort studies among people with diabetes in various countries [25]. Following the example of the original Diabetes MILES – Australia [25], the general aim of Diabetes MILES – The Netherlands was to gain greater insights into how people manage their diabetes and how it impacts on their lives.

In order to enable pooling of data for meaningful international comparisons and sub-group analyses, the Australian and Dutch MILES initiatives have both included a core set of measures focusing on key psychosocial and behavioural topics (e.g. self-care activities, symptoms of depression and anxiety, diabetes-related distress, illness perceptions, perceptions of insulin use) [25]. An important theme of mutual Australian and Dutch interest is diabetes self-care.

### Diabetes self-care

Most existing diabetes self-care questionnaires focus on the self-reported frequency of common behaviours or activities necessary for optimal management of diabetes [26,27]. While this is important, it provides a somewhat narrow perspective of how people manage their diabetes. One individual may always take the required number of insulin injections, eat healthily and be physically active, yet feel burdened or even burned out by his/her daily self-care efforts. Another person may decide to monitor his/her blood glucose levels only occasionally, as he/she feels that any incremental health benefits do not offset the loss in quality of life incurred by regular monitoring. Commonly used self-care inventories do not allow people with diabetes to indicate how important or how burdensome it is for them to undertake their daily self-care tasks. Furthermore, many were designed several years ago and lack some important dimensions of diabetes self-care (e.g. the use of cholesterol and blood pressure lowering drugs). A revised version of the Diabetes Self-Care Inventory [28] assessing the frequency, perceived importance and burden of a wide range of diabetes self-care activities was included in both the Australian and Dutch MILES surveys to enable psychometric validation in a large sample.

### Additional themes

Although the general survey themes were common between the Australian and Dutch studies, they differed in terms of several specific topics of interest, which were selected purposefully in each country. For Diabetes MILES – Australia, additional key topics include (1) empowerment and self-efficacy, (2) diabetes-specific quality of life, (3) optimism, (4) role of the family, (5) education and support programs and (6) hypoglycaemia unawareness. Topics specific to Diabetes MILES – The Netherlands are discussed below and include (1) subtypes of depression, (2) Type D personality, (3) mindfulness, (4) sleep and fatigue and (5) sexual functioning.

#### Subtypes of depression

To date, most research examining the general emotional well-being of people living with diabetes has focused on depression, with several studies showing that depressed individuals have higher healthcare use and expenditures, and are less likely to follow self-care recommendations [18,29]. In addition, depression in diabetes is associated with sub-optimal glycaemic control, the development of micro- and macro-vascular complications, and (all-cause) mortality [19,30-32]. However, it is unknown whether individuals experiencing specific symptom clusters or depression subtypes (e.g. somatic versus cognitive symptoms, melancholic versus atypical symptoms, dysphoria versus anhedonia, and depressive symptoms combined with anxiety or manic symptomatology) are particularly at risk for suboptimal health or self-care activities. Preliminary evidence supports the notion that within the spectrum of depression, symptoms with an emphasis on reduced positive affect (anhedonia) are associated with suboptimal glycaemic control, while symptoms related to negative emotions (dysphoria, anxiety) are not [33]. These findings warrant further study. A focus on the various symptoms and subtypes of depression may add to a better understanding of the relationship between depression and adverse health outcomes. This approach is well aligned with current research trends in psychiatry and cardiology [34,35].

#### Type D “distressed” personality

While depression has been associated with adverse health outcomes in people with diabetes [19,30-32], less is known about the risk imposed by other general forms of psychological distress [36]. In recent years, the Type D or “distressed” personality has emerged as a risk factor for adverse psychological and medical outcomes in people with cardiovascular and non-cardiovascular conditions [36,37]. Individuals with a Type D personality tend to experience negative emotions across time and situations (trait Negative Affectivity), but are inclined to inhibit self-expression in order to avoid disapproval or rejection by others (trait Social Inhibition) [38]. One potential mechanism through which Type D personality might exert a negative influence on health includes suboptimal self-care behaviour. The studies conducted so far have involved a variety of populations (healthy young adults, community samples, and people at high risk of cardiovascular conditions or those with established cardiac disease), and indicate that individuals with a Type D personality are less like to engage in physical activity and healthy eating, less likely to follow recommended medication regimens, and are less likely to seek consultations with health professionals when needed [39-44]. The only study to date addressing the health risks of Type D personality in diabetes populations demonstrated that people with type 2 diabetes and Type D personality did not differ in vascular history or physiological risk factors compared to their non-Type D counterparts [45]. However, Type D personality was related to a more sedentary lifestyle in women [45]. Furthermore, all participants with Type D personality experienced less social support and more stressful life events, loneliness, and emotional distress [45], which may interfere with optimal self-care [46,47].

#### Mindfulness

Another topic currently receiving increased research interest in psychosomatic research is mindfulness, a state of mind in which an individual intends to maintain awareness on the present moment in a nonjudgmental and open manner [48]. The goal of mindfulness interventions is to develop or increase a greater sense of emotional balance and well-being by disengaging oneself from strong attachment to beliefs, thoughts, or emotions [48]. While preliminary results have shown that mindfulness-based interventions can reduce emotional distress, and improve self-care and glycaemic control in individuals with diabetes [49,50], less is known about the association between mindfulness as a general personal attribute and problems with self-management and emotional well-being in people with diabetes.

#### Sleep

Previous studies have shown that the association between diabetes and sleep impairment is likely to be bi-directional [51]. Sleep disorders are a risk factor for the development of type 2 diabetes, and they exacerbate metabolic control in both type 1 and type 2 diabetes [51]. In addition, diabetes itself (especially when accompanied by suboptimal glycaemic control and overweight) is often followed by sleep disturbances [51-53]. Little is known, however, about the factors associated with sleep quality and quantity in people with diabetes, or about the relationship between sleep problems and self-care. Physical discomfort (including nocturnal hypoglycaemia, rapid changes in glucose levels, neuropathic pain, sleep apnoea) are likely to be implicated [51] but there may also be a link with emotional distress.

#### Sexual functioning

Sexual problems appear to be a common reality for people living with diabetes [54]. Until relatively recently, most studies had focused primarily on sexual difficulties in men, in particular erectile dysfunction [55]. However, this does not provide a complete account of sexual functioning in diabetes, as both men and women may face difficulties with respect to desire, orgasmic capacity or pain, in addition to arousal-related problems [56,57]. Furthermore, the majority of studies examining the presence of sexual dysfunction in people with diabetes do not address the level of intra- or interpersonal concern or distress these dysfunctions may cause [57]. We currently lack a large scale study that (a) includes both men and women with type 1 or type 2 diabetes from the same source population; (b) covers multiple domains of sexual functioning; (c) incorporates distress in the definition of sexual dysfunction; while it also (d) examines both physiological and psychosocial correlates of these problems.

### Research questions

Research questions to be addressed by Diabetes MILES – The Netherlands include (but are not limited to):

–  Is the Diabetes Self-Care Inventory - Revised a valid self-report measure of the frequency, perceived importance, and burden of diabetes self-care activities in people with diabetes?

–  Are different subtypes of emotional distress (melancholic versus atypical depressive symptoms, mixed depression/anxiety, anhedonia, manic symptoms) differentially associated with diabetes self-care activities and healthcare consumption?

–  Are people with Type D personality, compared to those without Type D personality (a) less likely to engage in adequate self-care activities in terms of diet, physical activity, medication use, disease monitoring, smoking and alcohol consumption; and (b) less likely to attend medical appointments or to consult their physician or nurse in case of symptoms or problems with diabetes self-care?

–  Is a higher level of mindfulness associated with greater engagement in self-care activities and better emotional well-being in people with diabetes?

–  Which factors are associated with sleep quality and quantity in people with diabetes? Is there a relationship between sleep problems and diabetes self-care?

–  What are the correlates of sexual dysfunction in people with diabetes?

## Methods/design

### Study design

Diabetes MILES – The Netherlands was designed as a national observational study of Dutch people with diabetes. Initially developed as an independent research project, the Dutch initiative was joined with its Australian counterpart to form The Diabetes MILES Study International Collaborative during the final stages of its survey development due to similarities in study design and goals. As a consequence, there are some differences between the two surveys in terms of key themes (discussed above) and procedures. One of these relates to the mode of data collection. While Diabetes MILES – Australia included both postal and internet-based versions [25], the Dutch survey was available online only. The online survey was created using the web application “SpITS Questionnaire”, which was developed by the local information technology service of the School of Social and Behavioural Sciences of Tilburg University. Questions were presented successively, with pre-programmed skip patterns for items that were not applicable (e.g. after a participant indicates that they do not use insulin, the questions relating to insulin use are skipped). To reduce the number of missing values, most items had forced choice response formats. Based on pilot testing, estimated completion time of the survey was 45 min. Participants were offered the possibility of completing the survey in multiple stages. In order to reduce participant burden, the survey consisted of a main questionnaire (items to be completed by all respondents) and five complementary modules. Each respondent first completed the main set of questions and was subsequently assigned randomly to one of the complementary modules by a computer algorithm. The survey was available for completion online from September 6^th^ to October 31^st^ 2011.

### Participants

In close co-operation with the Dutch Diabetes Association (DVN, Diabetes Vereniging Nederland), Dutch adults with diabetes were invited to participate in the online survey. Although we tried to reach non-members of DVN as well, most recruitment efforts focused on people registered with DVN. While people with self-reported diabetes of any type were allowed to complete the survey, the main analyses will focus on those with type 1 or type 2 diabetes. As individuals aged 18 years were approached for participation in a parallel Dutch MILES initiative among children and adolescents with diabetes, the lower age limit of the adult study was 19 years. No upper age limit was specified.

### Procedure

The study was advertised through several media channels, including Dutch health websites, the monthly DVN magazine ‘Diabc’, a DVN twitter account and a digital e-newsletter sent to all subscribed members of DVN and the Dutch Diabetes Research Foundation (Diabetesfonds). As an incentive for participation, participants were invited to enter a draw to win one of two tablet computers. Interested individuals were invited to visit the study’s website to find out more about taking part in the study. In addition to information about the aims, contents and design of the study, the website contained a page where people with diabetes aged 19 and older could register for the study. This page clearly stated that participation was voluntary and that all analyses and publications would be based on anonymous data. Participants provided an email address so that they could be sent a personalised link to access the online questionnaire. It was emphasised that this personal information would be used for this purpose only and, to ensure anonymity, no other personally identifiable information (e.g. name, home address) was requested. Digital informed consent was sought by asking participants to tick a box in front of a statement specifying that they had diabetes, were 19 years or older, had read and understood the study information on the registration page, and were willing to participate in the survey. After registration, every participant was assigned a unique identifier (ID number). This coding was generated automatically by the software and consisted of unique successive numbers. The software automatically checked whether a certain email address was already registered, thereby minimizing the possibility of someone registering and completing the survey twice. If participants had any questions, they were referred to the Frequently Asked Questions page of the study’s website. In addition, they could contact a member of the research team by telephone or email. The online survey responses were saved on a secure Tilburg University server and were exported to Microsoft Office Excel and SPSS (Statistical Package for Social Sciences) Version 18 (IBM SPSS Statistics, NY, USA) files for data cleaning and analyses.

### Measures

Selection of study topics and corresponding assessment instruments was based on literature review, consultation with experts in the field, and feedback from people with diabetes who had participated in previous surveys. For certain topics, no suitable pre-existing questionnaires were available, so appropriate study-specific items were designed by the research team. The main survey included standardised measures of general health, healthcare consumption, self-care behaviours and emotional well-being as well as items eliciting demographics and clinical characteristics. The five additional survey modules covered a wide range of topics, including (1) self-care attitudes and beliefs (e.g. psychological insulin resistance, fear of hypoglycemia); (2) subtypes of depression; (3) mindfulness and positive mental health; (4) sleep, fatigue and pain; (5) relationships with others (e.g. social support, sexual problems). An overview of the concepts and measures included in Diabetes MILES – The Netherlands is provided in Table 1.

**Table 1:** Variables included in Diabetes MILES – The Netherlands (2011)

**Concept**	Measure or variable *	**Survey version**
Demographics	Sex†‡, age†‡, marital status†‡, ethnicity†, education†‡, current employment†‡, shift work	All participants
Diabetes (clinical)	Diabetes type†‡, diabetes duration†‡, current treatment regimen†‡, current blood glucose level, most recent HbA_1c_†‡, HbA_1c_ target level, number of severe hypos/hypers in past year, main diabetes health professional, membership of patient organisation†‡	All participants
General health	Height†‡, weight†‡, waist circumference†‡, hip circumference, presence of and medication for co-morbid conditions (including diabetes complications)†‡, number of hospitalisations in past year	All participants
Health consultations	Number of contacts with health professionals† in past year, cancelled appointments in past year, consultation behaviour (10 items)	All participants
Self-care behaviours	Diabetes Self-Care Inventory Revised (unpublished) plus Diabetes MILES – Australia smoking items†‡	All participants
Medication taking	ASK-12: Adherence Starts with Knowledge 12-item version [61]	All participants
Physical Activity	IPAQ: International Physical Activity Questionnaire (Short Form) [62], questions about sedentary behaviour adapted from Diabetes MILES - Australia	All participants
Eating behaviour	38-item food frequency questionnaire	All participants
Disordered eating	6 items†‡ plus 2 items with respect to insulin adapted from Diabetes MILES – Australia	All participants
Alcohol consumption	1 item: number of units per week	All participants
Diabetes-related distress	PAID: Problem Areas In Diabetes scale [63]†‡	All participants
Depression	PHQ-9: Patient Health Questionnaire 9-item scale [64]†‡	All participants
Anxiety	GAD-7: Generalized Anxiety Disorder 7-item scale [65]†‡	All participants
Type D personality	DS14: Type D Scale-14 [38]	All participants
Stressful life event(s)	1 item: stressful life event(s) in past year	All participants
Loneliness	1 item: loneliness in past year	All participants
Psychological insulin resistance	ITAS: Insulin Treatment Appraisal Scale [66]†‡	Module 1
Fear of hypoglycaemia	HFS-II: Hypoglycaemia Fear Survey-II [67]	Module 1
Diabetes-specific avoidance	6 items	Module 1
Eating style	DEBQ: Dutch Eating Behavior Questionnaire [68]	Module 1,3
Beliefs about diabetes	BIPQ: Brief Illness Perception Questionnaire (diabetes version) [69]†‡	Module 1,3
Anhedonia	4-item anhedonia subscale HADS: Hospital Anxiety and Depression Scale [70]	Module 2
Manic symptoms	MDQ-NL: Mood Disorder Questionnaire [71]	Module 2
Subtypes of depression	IDS-SR: Inventory of Depressive Symptomatology – self reported [72]	Module 2
Mindfulness	FFMQ-NL: Five Facet Mindfulness Questionnaire – Dutch version [73]	Module 3
Neuropathic pain	NeuroQoL: Neuropathy and foot ulcer-specific Quality of Life questionnaire (physical symptom measures only) [74]	Module 3,4
Positive mental health	MHC-SF: Mental Health Continuum - Short Form (emotional and psychological subscales only) [75]	Module 3
Sleep	PSQI: Pittsburgh Sleep Quality Index [76]	Module 4
Fatigue	FAS: Fatigue Assessment Scale [77]	Module 4
Daytime sleepiness	ESS: Epworth Sleepiness Scale [78]	Module 4
Social support	MSPSS: Multidimensional Scale of Perceived Social Support [79]	Module 5
Relationship adjustment	DAS: Dyadic Adjustment Scale [80]	Module 5
Sexual problems	SSFS: Short Sexual Functioning Scale – male and female version (Enzlin et al., unpublished)	Module 5

* Unless otherwise specified, the question(s) was/were designed by the Diabetes MILES – The Netherlands research team.

† Diabetes MILES Study core measures, to be included in all surveys of adults living with type 1 or type 2 diabetes.

‡ Measures present in the pooled Australian-Dutch data-set.

### Ethical principles

The study protocol of Diabetes MILES – The Netherlands was approved by the Psychological Research Ethics Committee of Tilburg University, The Netherlands (EC-2011 5). As the study was internet-based, digital informed consent was obtained from all participants.

### Statistical analyses

Statistical analyses will be performed using the Statistical Package for Social Sciences (SPSS). Unless otherwise specified, a significance level of p<0.05 will be adopted in all statistical analyses. Sample and sub-sample characteristics will be presented using frequencies for categorical variables and mean ± standard deviation for continuous variables. Depending on the research question, differences between subgroups (e.g. men versus women, type 1 versus type 2 diabetes) will be tested using chi-squared tests for categorical data, and independent samples t-tests/analyses of variance for continuous variables. Multiple linear (continuous dependent variable) and logistic (binomial dependent variable) regression analyses will be used to study the association between the independent and dependent variables of interest. Other analyses may be applied and will be reported in the papers concerned. We aimed to recruit at least 3,000 participants in order to ensure that the study was powered sufficiently to enable various sub-group analyses.

### Sample characteristics

**Figure 1:**
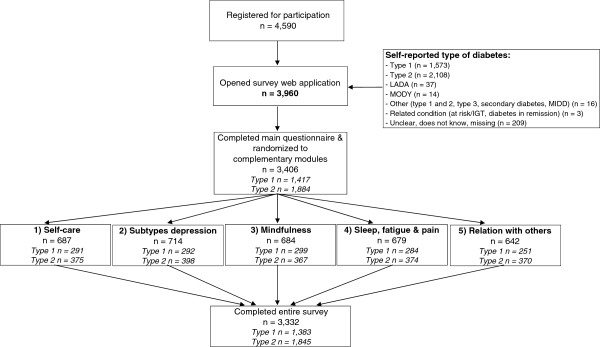
Flow chart of Diabetes MILES – The Netherlands.

Figure 1 provides an overview of study participation and drop-out. A total of 4,590 people registered an email address on the study website expressing interest in participation, of whom 86% (n=3,960) accessed the survey via the personal hyperlink. The latter number, reflecting all individuals who commenced the online survey (not necessarily completing it), defines the total Diabetes MILES – The Netherlands sample. We were not able to determine the reasons why the remaining 630 did not open the survey. Of the total sample (n=3,960; 100%), 1,573 (40%) self-reported having type 1 diabetes, 2,108 (53%) type 2 diabetes, and 70 (2%) a different type of diabetes or a related condition, e.g. LADA (Latent Autoimmune Diabetes in Adults), MODY (Maturity-Onset Diabetes of the Young), pre-diabetes, diabetes in remission. The remaining 5% of participants did not complete this item or their diagnosis was unclear to them. In total, 3,406 participants (86%) completed the main survey and were randomised to one of the five complementary modules (19-21% randomised to each module). The entire survey (i.e. the main survey plus one module) was completed by 3,332 participants (84%). Table 2 shows the demographic and clinical characteristics of the total Diabetes MILES – The Netherlands sample (n=3,960), and separately for participants with self-reported type 1 and type 2 diabetes. While the total sample showed a fairly equal sex distribution, women were slightly over-represented in the type 1 diabetes sub-sample. People with type 1 diabetes were younger, more likely to have a higher educational level and paid employment, and had lived for longer with their condition than those with type 2 diabetes. They had a lower mean body mass index, but a somewhat less optimal mean HbA_1c_. Compared to the national diabetes population distribution [58], those with self-reported type 1 diabetes, type 1 diabetes on insulin pump therapy, and type 2 diabetes using insulin were over-represented.

**Table 2:** Sample characteristics of Diabetes MILES – The Netherlands (2011)

	**All**	**Type 1 diabetes**	**Type 2 diabetes**
	**(n=3,960)**	**(n=1,573)**	**(n=2,108)**
**Demographics**			
Women	54% (2,133/3,925)	61% (958/1,572)	48% (1,020/2,108) ‡
Age in years	55 ± 14	47 ± 15	62 ± 10 ‡
Ethnic minority	3% (104/3,884)	2% (34/1,573)	3% (58/2,108)
Educational level			
Low	27% (1,040/3,870)	19% (293/1,571)	33% (684/2,101)
Middle	34% (1,313/3,870)	35% (546/1,571)	34% (704/2,101)
High	39% (1,517/3,870)	47% (732/1,571)	34% (713/2,101) ‡
Having a partner	80% (3,107/3,889)	80% (1,260/1,573)	80% (1,682/2,108)
Paid employment	48% (1,838/3,873)	63% (992/1,571)	36% (763/2,104) ‡
**Clinical characteristics**			
Duration of diabetes in years	16 ± 13	23 ± 15	11 ± 8 ‡
Diabetes treatment			
Insulin pump	24% (910/3,786)	48% (761/1,572)	6% (125/2,102) ‡
Insulin injections	50% (1,891/3,786)	53% (830/1,572)	47% (995/2,102) †
Glucagon-like peptide-1 (GLP-1) agonist injections	2% (62/3,786)	0.1% (2/1,572)	3% (59/2,102) ‡
Blood glucose lowering tablets	44% (1,627/3,664)	7% (111/1,566)	74% (1,474/1,987) ‡
Lifestyle only	3% (99/3,726)	0% (0/1,572)	5% (95/2,042) ‡
Most recent HbA_1c_ (mmol/mol)	56 ± 12	58 ± 12	54 ± 12 ‡
Body Mass Index (kg/m^2^)	28 ± 6	25 ± 5	30 ± 6 ‡
Macro-vascular disease and/or micro-vascular complications *			
None	69% (2,544/3,691)	70% (1,077/1,539)	68% (1,383/2,041)
One	20% (729/3,691)	18% (276/1,539)	21% (436/2,041)
Two or more	11% (418/3,691)	12% (186/1,539)	11% (222/2,041) †

Values are mean ± standard deviation, unless otherwise specified.

* Self-reported myocardial infarction, stroke, peripheral arterial disease, nephropathy, retinopathy, neuropathy and/or foot condition.

† Difference between self-reported type 1 and type 2 diabetes *p* < 0.05.

‡ Difference between self-reported type 1 and type 2 diabetes *p* < 0.001.

## Discussion

Diabetes is a common long-term condition, and as the prevalence for all types of diabetes is likely to continue to increase during the next two decades [1,5,59], it has never been more vital to further our understanding of the everyday experiences, well-being, self-care activities, and health beliefs of people living with this condition. By examining the way people manage their diabetes, and the physical, emotional and social difficulties they encounter, unmet needs can potentially be identified and used to inform care provision. The results of Diabetes MILES – The Netherlands may provide insights into which subgroups of people are at high risk of problems with self-management and emotional well-being, and could guide the development of future intervention studies.

### Strengths and limitations

The major strengths of Diabetes MILES – The Netherlands include the relatively large sample size (n=3,960) and the wealth of detailed data captured regarding well-being, self-care and health. The present Diabetes MILES – The Netherlands study may serve as the baseline assessment of a potential longitudinal cohort study examining prospective associations between emotional well-being and other health outcomes. Under the umbrella of The Diabetes MILES Study International Collaborative, analysis of the pooled Dutch and Australian Diabetes MILES datasets is currently underway. This pooled dataset has a total participant sample of 7,019, which is large enough to permit sub-group analyses of rare groups within the sample, and thorough examination of less common characteristics.

The limitations of Diabetes MILES – The Netherlands are those inherent to any internet-based self-report survey. By advertising the study in relevant health media rather than contacting a pre-determined random sample, those who are actively engaged in their diabetes care, seek out opportunities to increase their knowledge or communicate with peers, or for whom diabetes is explicitly present in their daily lives are likely to be over-represented. This may be reflected by the fact that the majority of our sample consisted of those with self-reported type 1 diabetes or type 2 diabetes using insulin therapy, while the vast majority of people with diabetes have type 2 diabetes managed with a combination of lifestyle modifications and blood glucose lowering tablets. Furthermore, over 90% indicated that they were members of DVN. This is unsurprising given DVN’s prominent role in advertising for the study. With respect to prevalence estimates, however, this may limit the generalisability of our findings to the general Dutch diabetes population.

While the decision to offer the survey for online completion only may have introduced some bias into the sample, the impact of this is unlikely to be substantial. Recent figures from the Dutch Central Bureau of Statistics show that The Netherlands is among the countries with the highest internet coverage rate in Europe, with over 90% of the Dutch population having access to the internet [60]. The main reasons for not having internet access include lack of interest (3%) and insufficient knowledge/physical disabilities (1%) [60]. With 56% of participants in the present study aged between 50 and 70 years, 12% aged over 70 years, and an overall age range spanning 19 to 90 years, older adults did not appear to be deterred from participating in an online study. However, as 69% of the total sample did not report having micro-vascular complications or macro-vascular disease (Table 2), those in relatively good health may have been somewhat over-represented.

As all clinical variables were determined through self-report, we cannot rule out a certain margin of error in these measures. For example, some people with type 2 diabetes using insulin treatment may have self-identified as having type 1 diabetes, while for self-reported complications or co-morbidities, some people may not be aware of specific diagnoses. For measures susceptible to bias through social desirability (e.g. most recent HbA_1c_, weight, waist-hip measurements), we hope that our procedures to ensure anonymity minimised some of these effects.

Although people with self-reported diabetes of any type were invited to complete the survey, only a small minority (n=70) indicated having a type of diabetes other than type 1 or type 2, or a condition closely related to, but not actually, diabetes. While this limits the opportunity to compare outcomes across less common diabetes types (e.g. MODY, LADA, secondary), these findings inform us about which individuals self-identify with a study focusing on “living with diabetes”. We recommend that future research efforts target these minority types of diabetes to ensure greater understanding of the specific well-being and self-care needs of these groups.

By definition, we were unable to include people with type 2 diabetes who are unaware of their condition (that is, those with undiagnosed type 2 diabetes). Epidemiological studies have shown that up to 50% of all Dutch people with diabetes are undiagnosed [59], though more recent estimates suggest that the number of Dutch people with undiagnosed type 2 diabetes has decreased to approximately one quarter of the total diabetes population, possibly due to improvements in screening and early diagnosis [6].

People from ethnic minority backgrounds were under-represented in our sample. Due to practical considerations, the Diabetes MILES – The Netherlands survey was available only in Dutch. Knowing that ethnic minorities represent a vulnerable subgroup in terms of their health outcomes, future MILES initiatives need to promote participation of people from culturally and linguistically diverse backgrounds [25].

Although the breadth of the survey enables a thorough analysis of the psychosocial wellbeing of participants, the length of the survey (estimated completion time 45 min) may have caused individuals with mental health co-morbidities or physical disabilities to leave the study prematurely, or not to enter the study in the first place.

Taking into account that it was impossible to register for the study twice with the same email address, we may have systematically excluded participants’ family members also diagnosed with diabetes (and thereby eligible) but using the same email address. It is also possible, although highly unlikely, that individuals may have participated multiple times using different email addresses.

## Conclusions

Diabetes MILES initiatives are currently being planned in other countries, and this will facilitate further data pooling as well as provide specific insights into what it is like to live with diabetes in various countries. Those interested in joining The Diabetes MILES Study International Collaborative are invited to contact Professor Jane Speight or Professor François Pouwer. Even though the main focus of The Diabetes MILES Study is currently on adults with type 1 or type 2 diabetes, future research is expected to also include children and adolescents with diabetes and their parents, and special subgroups of diabetes (e.g. gestational, MODY, LADA, secondary), in order to provide a more comprehensive overview of the psychosocial implications of living with diabetes.

## Competing interests

The authors declare that they have no competing interests.

## Authors’ contributions

JS conceived the Diabetes MILES Study and together with FP developed The Diabetes MILES Study International Collaborative. FP is principal investigator of Diabetes MILES – The Netherlands; GN and MB contributed to the development of survey content, managed the survey data collection, and conducted data cleaning and analyses. GN and FP produced the first draft of this manuscript; MB, JB and JS contributed substantially to its development. All authors approved the final version.

## Pre-publication history

The pre-publication history for this paper can be accessed here:

http://www.biomedcentral.com/1471-2458/12/925/prepub
